# Living Organ Donation: An Ethical Evolution or Evolution of Ethics?

**Published:** 2010-05-01

**Authors:** N. Ghahramani

**Affiliations:** *Division of Nephrology, Pennsylvania State University College of Medicine, USA*

**Keywords:** Kidney Transplantation, Medical ethics, Renal disease

## Abstract

The disparity between available and needed organs is rapidly increasing, and the number of patients dying while still on the waiting list is growing exponentially. As a partial solution to this disparity, living unrelated transplantation is being performed more frequently, and some have proposed providing financial incentives to donors. The aim of this discussion is to illustrate that with an ever-increasing number of living unrelated transplantations, society and the transplant community should adopt a more active role in developing specific strategies to scrutinize the process. The current paper will also examine the viewpoint that medical ethics is not separable from the prevailing needs of society and involves a constant balancing of often opposing goods. Issues surrounding living unrelated donor transplantation illustrate ethics as a dynamically evolving field, which is often influenced by necessity and which evolves with progression of science and society. As part of this evolution, it is the collective responsibility of society and the transplant community to devise safeguards to guarantee adherence to basic principles of ethics and to avoid “situational ethics.”

## INTRODUCTION

Following the first successful kidney transplantation in 1954, and the subsequent improvements in immunosuppressant medications, the major barriers against solid organ transplantation seemed to have been overcome. This set the stage for subsequent discoveries, innovations and improvements in surgical techniques towards the development of strategies for transplantation of other solid organs including the liver, heart, lungs and pancreas. The initial excitement has been overshadowed by the recognition of organ shortage and the need for improved allocation. The exponentially increasing number of patients on the waiting list and the relative constancy of the donor pool have translated into the death of approximately 12 patients on the waiting list each day in the United Sates [1]. The disparity between available and needed organs is growing rapidly, and the number of patients dying while still on the waiting list is growing exponentially. Transplantation has moved from being just another form of medical advance to a matter of public policy, taking efficiency and equity into account. The concepts of brain death definition, cadaveric organ procurement, as well as fair and just allocation of organs have all been debated topics of discussion among bioethicists, religious scholars and social scientists. Transplantation has opened various other discussions of ethical concern, such as financial incentives for living donation, donation by inmates, minor sibling-to-sibling organ donation, stem cell transplantation and xenotransplantation. The current discussion will focus on the first topic, using the kidney as an example. The aim of this discussion is to illustrate that with an ever-increasing number of living unrelated transplantations, society and the transplant community should adopt a more active role in developing specific strategies to scrutinize the process. A secondary objective is to examine the viewpoint that medical ethics is not separable from the prevailing needs of society and that it involves a constant balancing of often opposing “goods.”

## AN OVERVIEW OF THE CURRENT PRACTICE

As a transplant physician who has practiced in a developing country for 10 years and in the US for nearly nine years, the author has often witnessed ethical dilemmas faced by patients, families and physicians regarding various aspects of transplantation. For many years, while living unrelated donor transplantation (LURT) was being performed in many developing nations, the medical community in the industrialized world, and particularly in the US, had been reluctant to discuss this as a feasible form of transplantation. The main reasons for this reluctance were the valid concerns about risks to the donor, as well as fears of commercialization and exploitation. Transplant physicians who presented data including LURT often encountered arrogant objection, and discussion of LURT at scientific meetings was considered a taboo. However, in recent years, with the increasing demand for organs, it has become clear that the combination of deceased and living related donations cannot provide sufficient organs for transplantation and that many patients are dying on a daily basis while awaiting transplantation. Various centers in the industrialized nations, including the US, have become increasingly lenient towards LURT, as evidenced by a six-fold increase in the percentage of LURT (including spouses) from 1988 to 2003 [2].

Transplant centers have developed algorithms—mainly through psychosocial assessments—to safeguard rights of the parties involved, ensuring that the motive for donation is altruistic and that there is no intimidation or commercialization involved. The donors in these situations are usually spouses, close friends, colleagues and occasionally anonymous. There are several potential problems associated with this scheme. The definition of a “close friend” is rather arbitrary, relying quite subjectively on the interpreter’s judgment. The scheme assumes that the spouse, friend or colleague are (or should be) acting altruistically, often reflecting the interpreter’s own ethics, viewpoints and attitudes. While in most instances this might certainly be the case, coercion and subtle intimidation are often very difficult to determine in a 45-minute interview. Furthermore, there are concerns about “conflict of interests”, particularly when a colleague (sometimes an employee) volunteers to donate an organ to another colleague.

## STAKEHOLDERS IN LIVING DONOR TRANSPLANTATION

The principal stakeholders in the living organ donation scheme include the patient (recipient), the donor, their families, society and the medical community. The premises of the present discussion are as follows:

The recipient has the right to pursue improvement in quality of life, life expectancy and minimization of suffering.

The donor has the right and autonomy to donate an organ in an informed manner, with minimal suffering or detriment to his/her health.

The families of the donor and recipient have the right to pursue a meaningful life with minimal disruption. 

The medical profession has the obligation to provide the donor and recipient with the required information regarding risks and benefits of the treatments and to pursue a course of action consistent with *primum non nocere*—the fundamental medical principle of “first do no harm”—thus, the medical profession has the right to refuse a treatment determined to be harmful to one of the parties involved. Potential harm is not limited to bodily harm, but also harm to the personal integrity of the recipient or the donor. The profession also has the right to be appropriately compensated for the services provided.

The society has the right to prevent any action that violates laws and has the responsibility to provide means for the reasonable achievement of the wishes of the parties involved, safeguarding efficiency and equity, and avoiding exploitation of the parties involved. If we assume that the individual patient is the collective responsibility of society, it would appear that the donor is, at least partially, acting in a way to fulfil the duty of society.

## LIVING UNRELATED TRANSPLANTATION AND FINANCIAL COMPENSATION

It is clear that if an organ donation is harmful and life threatening to the donor, we should not even consider taking it from a consenting living donor, whether related or unrelated. The scientific community has determined that donation of a kidney is not life threatening, however, it is not without harm. There are the potential harms of the surgical procedure and those associated with living with a single kidney [3-7]. However, the potential benefit to the recipient significantly outweighs the potential harms to the donor, assuming that the donor is an adult who is mentally competent and has been adequately informed about the risks. On the other hand, there is minimal benefit to the donor and thus, it has traditionally been expected that the donor will engage in such a contract solely on the basis of altruistic motivation.

With related donor transplantation, altruism is the expected driving force; however, regarding unrelated donors, several valid questions have been raised. Why should the unrelated donors not be at least partially rewarded for their donation? Why should they be expected to undergo the surgery and live with one less organ for the rest of their lives? Are the other parties involved (physicians, surgeons, nurses, *etc.*) providing their services only altruistically? Why should the only individuals sacrificing their bodies not be appropriately acknowledged? Although current laws in most countries and guidelines by professional societies [8] prohibit the sales of organs, it has been debated that provision of financial incentive seems not only fair, but may also encourage donation and subsequently benefit the patients on the waiting list.

The main opponents of providing financial incentives have voiced concern over “devaluing” the body to a mere commodity and the potential for commercialization [9]. The remainder of the discussion will focus on the latter. Regarding the former, suffice it to note that some would argue that the body is a property and, in fact, the most valuable commodity that an individual possesses. They would contend that the owner of this property has a right to sell part of it for his/her better good [10].

## COMMERCIALIZATION OF ORGANS

Some have proposed a market for organ donation or sale. The proponents of this model propose a legitimate governmental or non-profit non-governmental organization to take charge for the responsibility of compensating the donor, without any direct contact between donors and recipients [11, 12]. This would eliminate profit-seeking middlemen and organ brokers. While in certain instances, this practice has led to elimination of the waiting list [13], evidence for negative impact of kidney donation for the donors have been reported [14].

In addition to direct payment, various other forms of compensation such as life and health insurance, medal of honor, reimbursement for travel expenses, compensation for time out of work, or a tax credit have been proposed [15]. The potential problem with this model is that if it is not well organized, it will open the door to an organ market, where the organs are sold to the highest bidder, benefiting the rich and disadvantaging the poor. Concern has also been raised that this will reduce altruistic kidney donation and discourage deceased multi-organ donation [16]. However, some believe that it does not preclude increased donation [17], and others have shown that it has not inhibited the establishment of deceased donor transplantation programs [11].

Opponents to any form of compensation and an organ market [9, 18, 19] cite the concern that the poor will be viewed as mere providers of spare parts and will live with fewer organs, adding this to their list of disadvantages. According to this viewpoint, the market will be driven by poverty and the poor will be at a disadvantage compared to the wealthier, feeling a disproportionately higher pressure to sell their organs [9]. On a global scale, this could translate into people from rich nations travelling to poor countries to buy organs. There is the concern that the market could potentially lead to demeaning bodies to “articles of trade,” degrading human relationships, and particularly damaging the altruistic bond [9]. There is also the concern about the occasional coercion of a spouse by an addicted spouse into selling an organ to pay for the addiction. 

The question of whether or not the financial compensation leads to an improvement in the life of the donor has been addressed in several studies. There is evidence that with rampant commercialization, the benefits to the donor are minimal and much of the “financial reward” is drained by brokers [20]. In such settings, analysis has determined that the donors are actually not better off [21]. 

Although it is practically impossible to calculate all the risks and benefits in an economical model, it is clear that there are some potential benefits to providing financial incentives for organ donation. The same analysis that concludes that paid donors are not better off, has noted that most of the money received by donors are spent on the essentials: debts, food, and clothing [21]. Some potentially common scenarios in favor of providing incentive to donors are: a parent who donates an organ with the motivation of providing the costs of treatment of a sick child or paying for a child’s education; or the head of household who sells a kidney to buy a plot of land to provide for the family. 

The opponents who fear the evils associated with rampant commercialization tend to understate society’s responsibility. In response to their fears and concerns, it should be noted that society has to play an active vigilant role in safeguarding the individual’s rights, rather than categorically prohibiting any forms of incentive in fear of potential exploitation. Which one is more unethical? A donor selling an organ in desperation for the better good of self and/or family or the society refusing to acknowledge and find means to alleviate the desperation and misery of the individual. Similarly, is it more unethical for the recipient to reward a potential donor with monetary incentive or for the society not to have better plans for relieving his/her suffering? Which is more unethical? Transplantation from a paid volunteer unrelated donor or one from a living-related donor or spouse who is under some degree of family pressure or emotional coercion. It seems clear that intimidation of a related donor is no more ethical than rewarding a willing unrelated donor. Finally, is it more ethical to let patients die on the list or to compensate volunteer donors? In a similar argument, Veatch has proposed that it is time to lift the ban on marketing organs and considers it a moral necessity as a “lesser moral evil” [22]. 

## CONCLUDING REMARKS

There is little doubt that commercialization of organ donation is fraught with drawbacks, dangers and potential immoral consequences. On the other hand, it is clear that efforts to increase the rate of organ donation through education have failed and sole moral incentives have not worked. Organs are currently limited by supply, and in the hope of expanding the available organs, it seems prudent to provide incentives not only to encourage donation, but also in order to express appreciation. In the process, we should be cognizant of the fact that we might be sacrificing some goods for the sake of other potentially more meritorious goods, weighing the ethical and moral risks of one against the other. The obligation of society is to establish safeguards to protect all parties involved, as well as the humane inter-relationship between donor and recipient. In this regard, the method of acknowledging the good deeds of donors is of paramount importance and it is the author’s belief that this should not be the recipients’ responsibility, but rather the collective responsibility of society. Rather than resisting any changes, it is clear that we need to look for feasible, ethical alternatives to the current model. This is not limited to whether or not donors should be compensated. Now that LURT has become an ever increasing reality, society and the transplant community should devise safeguards to scrutinize the process. 

Discussions about financial incentives for organ donation have moved from being a taboo to a passionate controversy. While a few years ago, when proposed from outside the US, there would be severe objections to any form of compensated unrelated donation, now that it has been recognized as a feasible reality, authorities in the field are discussing potential means of making it work better, rather than totally condemning the thought. In conclusion, issues regarding LURT illustrate evolving ethics parallel to the needs of time. This demonstrates that bioethics is by no means a static field but rather a dynamic discipline, which is shaped by necessity and which evolves with evolution of science and society. As part of this evolution, it is the collective responsibility of society and the transplant community, in particular, to devise safeguards to guarantee adherence to basic principles of ethics, rather than to passively allow “situational ethics” to prevail. While acknowledging the inherent difficulties in establishing an optimal regulatory system that would be able to compete with the global black market, such a system seems essential to successfully prevent possible exploitations of the poor and the disadvantaged, particularly in developing countries. 
